# Electroacupuncture Attenuates Hepatic Ischemia-Reperfusion Injury by Modulating the Esr1/TAK1–JNK/p38 Signaling Pathway in Rats

**DOI:** 10.1155/mi/4932970

**Published:** 2025-08-30

**Authors:** Xiaofang Fan, Wei Guo, Xiaodan Yang, Hao Zhang, Bruno Fink, Lingyu Hu, Xiaoguang Wang

**Affiliations:** ^1^Faculty of Graduate Studies, Zhejiang Chinese Medical University, Hangzhou 310053, Zhejiang, China; ^2^Department of Surgery, The Second Affiliated Hospital of Jiaxing University, Jiaxing 314000, Zhejiang, China; ^3^Rhine-Nahr-Palatinate Heart and Vascular Center, Salinenstraße 35a, Bad Kreuznach 55543, Germany; ^4^Noxygen Science Transfer and Diagnostics GmbH., Lindenmatte 42, Elzach 79215, Germany; ^5^Department of Surgery, Jiaxing University Affiliated Hospital, The First Hospital of Jiaxing, No. 1882 South Central Road, Jiaxing 314000, Zhejiang, China

**Keywords:** electroacupuncture, Esr1 gene, hepatic ischemia-reperfusion injury, hepatocyte apoptosis, inflammation modulation, TAK1–JNK/p38 signaling pathway

## Abstract

Electroacupuncture (EA) has demonstrated protective effects against hepatic ischemia-reperfusion injury (HIRI) in rat models. This study aimed to explore the underlying molecular mechanisms by which EA exerts its protective effects against HIRI. Gene expression microarray data from the Gene Expression Omnibus (GEO) database were analyzed to identify genes associated with HIRI, followed by differential expression analysis. Our results revealed that EA treatment significantly reduced serum alanine aminotransferase (ALT) and aspartate aminotransferase (AST) levels, as well as myeloperoxidase (MPO) activity in liver tissues. Histological analysis indicated decreased necrotic areas and apoptosis in EA-treated liver tissues. Molecular assessments demonstrated that EA downregulated Esr1 expression and inhibited the activation of the TAK1–JNK/p38 signaling pathway, thereby reducing hepatocyte apoptosis and inflammatory responses. These findings suggest that EA serves as a potent therapeutic approach to alleviate HIRI by targeting the Esr1/TAK1–JNK/p38 signaling pathway.

## 1. Introduction

Hepatic ischemia-reperfusion injury (HIRI) represents a major clinical challenge that arises when a previously ischemic and hypoxic liver undergoes reoxygenation and reperfusion, initiating a cascade of pathological responses. These responses include an acute inflammatory response and excessive production of reactive oxygen species (ROS), leading to significant tissue damage and organ dysfunction [1–3]. Epidemiologically, HIRI is commonly observed in various clinical scenarios such as liver transplantation, hepatic resection, and ischemic liver diseases [4]. The pathogenesis of HIRI involves multiple factors, including oxygen deprivation, hypoxia-induced cellular damage, and subsequent reperfusion injury due to restored blood flow and disrupted microvascular integrity [5]. These disturbances can result in severe cellular, organ, and systemic dysfunction, potentially culminating in life-threatening conditions if unmanaged [6].

Traditional therapeutic strategies for HIRI primarily focus on modulating inflammatory mediators, utilizing antioxidants, and adopting specific surgical techniques such as hepatic portal vein blockade or selective hepatic perfusion to protect the ischemic liver tissue [7]. However, the limited efficacy of these interventions highlights the need for alternative and more effective therapeutic approaches.

Electroacupuncture (EA), a key modality of traditional Chinese medicine (TCM), has attracted growing interest for its clinical application. EA involves delivering electrical stimulation at specific acupoints to modulate the neuroendocrine and immune systems, offering therapeutic and preventive benefits against various diseases [8]. Extensive studies have documented its clinical utility in the management of pain, neurological disorders, and diseases involving the liver, heart, and kidneys [9]. Specifically, EA has shown promising results in mitigating HIRI in rat models by downregulating the expression of Esr1 and inhibiting the activation of the TAK1–JNK/p38 signaling pathway [10]. This finding provides new insight into the potential mechanisms by which EA benefits patients with HIRI, establishing a theoretical basis for broader clinical application [11]. Although the precise therapeutic mechanisms are still under investigation, the available evidence supports the significant potential of EA as an alternative treatment for HIRI [12].

This study aims to advance understanding of the molecular mechanisms through which EA at specific acupoints influences HIRI in rats. In addition, the research seeks to elucidate the therapeutic potential of EA, which could potentially improve its effectiveness and clinical application for the treatment of liver injuries.

## 2. Methods

### 2.1. Bioinformatics Analysis

The gene expression microarray GSE117915 associated with HIRI rats was obtained from the Gene Expression Omnibus (GEO) database (http://www.ncbi.nlm.nih.gov/geo). The dataset included 12 Sham-operated samples and 12 HIRI samples. Differentially expressed genes (DEGs) were identified using the “limma” package in R, with screening criteria set at |log fold change |logFC| > 0.17 and *p* value < 0.05.

### 2.2. HIRI Model Construction and Animal Grouping

Considering the high anatomical and hemodynamic similarity between rat and human livers, as well as the greater stability and reproducibility of rat models in HIRI studies, we selected the rat model for this study [13, 14]. A total of 80 male Sprague–Dawley rats (aged 6–8 weeks and weighing 180–200 g) were procured from Hunan SLAC Laboratory Animal Co., Ltd. The animals were housed individually in standard cages under controlled environmental conditions (temperature 22 ± 1°C; 12:12 h light/dark cycle), with free access to food and water. After a week of acclimatization, the rats were randomly divided into seven groups, with eight animals per group.

The HIRI model was established through a midline laparotomy, followed by the placement of nontraumatic vascular clamps on the portal vein and hepatic artery supplying the median and left lateral lobes of the liver, thereby inducing partial hepatic ischemia. After 1 h of occlusion, the clamps were gently removed to allow reperfusion for 12 h, resulting in approximately 70% HIRI in rats. In the Sham group, rats underwent laparotomy and vessel dissection without vascular clamping [15]. Adeno-associated viruses (AAVs) encoding sh-Esr1, sh-NC, oe-Esr1, or oe-NC (Gene Pharma, Shanghai, China) were administered via tail vein injection at a dose of 5 × 10^11^ viral genome copies in a 250 μL volume [16, 17], followed by the HIRI procedure [18]. In the TAK1 inhibitor group, rats received intraperitoneal injection of 5Z-7-oxozeaenol (5Z-7-ox; O9890-1 MG, Sigma, St. Louis, MO, USA) dissolved in DMSO at a dose of 5 mg/kg, 30 min prior to the ischemia-reperfusion procedure [19].

Acupoints were located at the depression anterior and inferior to the fibular head on the lateral side of the hind limbs of the rats. Sterile acupuncture needles were inserted to a depth of 2–3 mm and connected to an EA device (HANS-200E, Nanjing Jisheng Medical Technology Co., Ltd., Nanjing, China). Continuous stimulation was applied for 30 min at an intensity of 4 mA and a frequency of 2/100 Hz [20].

The experimental groups were as follows: Sham (Sham operation), HIRI (HIRI model), HIRI + EA (HIRI model with EA treatment), HIRI + sh-NC (HIRI model with sh-NC adenovirus injection), HIRI + sh-Esr1 (HIRI model with sh-Esr1 adenovirus injection), HIRI + oe-NC (HIRI model with oe-NC adenovirus injection), HIRI + EA + oe-NC (HIRI model with oe-NC adenovirus and EA treatment), HIRI + EA + oe-Esr1 (HIRI model with oe-Esr1 adenovirus and EA treatment), HIRI + EA + oe-Esr1+DMSO (HIRI model with oe-Esr1 adenovirus and DMSO and EA treatment), HIRI + EA + oe-Esr1 + 5Z-7-ox (HIRI model with oe-Esr1 adenovirus and 5 Z-7-ox and EA treatment). The terms “oe” and “sh” denote overexpression and short hairpin, respectively, while “NC” represents the negative control.

The in vivo animal experimental protocol was approved by the Animal Ethics Committee of Jiaxing University Medical College and conducted in accordance with the “Guidelines for the Care and Use of Laboratory Animals” published by the National Institutes of Health. Liver tissues were harvested for subsequent experiments and fixed in 4% paraformaldehyde for terminal deoxynucleotidyl transferase dUTP nick end labeling (TUNEL) staining and immunofluorescence-based immunohistochemistry.

### 2.3. Primary Isolation, Culture, and Transfection of Rat Hepatocytes

Primary hepatocytes were selected as the target cells in this study due to their critical role in HIRI. Hepatocytes represent the primary parenchymal cells directly affected during HIRI and play a key role in regulating the inflammatory response [21, 22]. Furthermore, damage-associated signals released from hepatocytes significantly influence the activation of nonparenchymal cells (e.g., macrophages) during the early inflammatory cascade. Liver tissue from a HIRI rat was aseptically collected and immersed in sterile D-Hanks solution, followed by two washes with PBS. The tissue was then transferred into high-glucose Dulbecco's modified eagle medium (DMEM; 11965126, Gibco), and the liver capsule was gently disrupted using ophthalmic forceps to release hepatocytes into the medium. The cell suspension was subsequently filtered through a 200-mesh stainless steel cell sieve and transferred into a sterile 15 mL centrifuge tube. After centrifugation at 800 rpm for 5 min, the supernatant was discarded. The cells were resuspended in high-glucose DMEM, filtered again through a 200-mesh sieve, and collected by a second centrifugation.

The cells were then resuspended in a high-glucose DMEM hepatocyte growth medium. The hepatocyte suspension was adjusted to a density of 5 × 10^5^ cells/mL and seeded into culture flasks pre-coated with rat tail collagen. The flasks were incubated at 37°C in a 5% CO_2_ incubator. After 24 h, the medium was replaced to remove nonadherent cells. The hepatocytes were maintained in high-glucose DMEM growth medium, with medium changes performed every other day. Viral transfection experiments were conducted after 3–4 passages.

For transfection, primary hepatocytes were seeded at a density of 5 × 10^5^ cells/mL in six-well plates and cultured overnight at 37°C. Cell confluence was adjusted to about 60% to 80% on the following day. Then, 10 µL of adenoviruses containing sh-Esr1-1, sh-Esr1-2, sh-Esr1-3, and sh-NC (1 × 10^8^ PFU; sequences listed in Supporting Information 1: Table S1) were individually added to the medium. Polybrene was simultaneously added at a final concentration of 10 µg/mL to enhance transduction efficiency. After 24 h, the medium was replaced with a fresh growth medium. Following infection, cells were cultured for 72 h prior to analysis. Reverse transcription-quantitative PCR (RT-qPCR) and western blotting were conducted to assess the efficiency of gene silencing. All adenoviruses used in the experiments were designed, synthesized, and supplied by Gene Pharma (Shanghai, China).

### 2.4. Assessment of Liver Damage and Cytokine Levels

Peripheral blood was collected from rats in each group, and serum was isolated to measure serum alanine aminotransferase (ALT) and aspartate aminotransferase (AST) levels using an automated chemical analyzer (AU5400; Olympus).

Liver tissue samples were fixed in 10% formalin, embedded in paraffin, and sectioned into 4–5 µm slices. Following deparaffinization, rehydration, and washing, the sections were stained with hematoxylin and eosin (H&E) [23]. The necrotic areas were examined and photographed under a microscope (Model BX63, Olympus). The percentage of the necrotic area was calculated by dividing the necrotic area by the total area examined and expressed as a percentage [24]. The histological severity of HIRI was evaluated according to the Suzuki scoring system (Supporting Information 1: Table S2).

Inflammatory status was evaluated by measuring the levels of cytokines TNF-α, IL-1β, and IL-6 in rat serum. Peripheral blood was collected from each group and serum was separated by centrifugation. Cytokine levels were quantified using enzyme-linked immunosorbent assay (ELISA) kits (Thermo Fisher Scientific) according to the manufacturer's instructions for TNF-α (#KRC3011C), IL-1β (#BMS630), and IL-6 (#BMS625).

### 2.5. Myeloperoxidase (MPO) Activity Assay in Liver Tissue

MPO activity in liver tissue was measured following a previously described protocol [25]. In brief, 100 mg of liver tissue was homogenized in 2 mL buffer containing 3.4 mmol/L KH_2_HPO_4_ and 16 mmol/L Na_2_HPO_4_ (pH = 7.4). The mixture was then centrifuged at 10,000 × *g* for 20 min at 4°C. The resulting pellet was resuspended in 10 volumes of buffer composed of 43.2 mmol/L KH_2_HPO_4_, 10 mmol/L EDTA, 6.5 mmol/L Na_2_HPO_4_, and 0.5% hexadecyltrimethylammonium (pH 6.0), followed by sonication for 10 s. The supernatant was mixed with 3,3',5,5'-tetramethylbenzidine and heated at 60°C for 2 h. The absorbance was then measured at 655 nm using a spectrophotometer [26].

### 2.6. TUNEL Staining

TUNEL staining was performed using the In Situ Cell Death Detection Kit (11684795910; Roche). In brief, paraffin-embedded liver sections were deparaffinized, rehydrated and washed three times with PBS for 5 min each. Proteinase K (1:9 dilution in PBS) was applied to cover the tissue and incubated at 37°C for 30 min, followed by three PBS washes. The sections were immersed in blocking solution (3% H_2_O_2_) at room temperature for 10 min and then, washed thrice with PBS. TUNEL reaction mixture was added to the samples and then, incubated in the dark at 37°C for 60 min. After three additional PBS washes, TUNEL-positive cells were imaged using a fluorescence microscope (DMi8; Leica Microsystems). Quantification was performed by analyzing five randomly selected fields per section and the percentage of TUNEL-positive cells was calculated as follows: (Number of TUNEL-positive cells/total number of cells) × 100%.

### 2.7. Immunofluorescence Staining

Paraffin-embedded liver sections (5 μm thickness) were subjected to immunofluorescence staining as previously described [27]. In brief, sections were incubated with 5% goat serum for 1 h to block nonspecific antibody binding. They were then incubated overnight at 4°C with primary antibodies: anti-CD68 (1:50, ab201340, Abcam) and anti-GR-1 (1:20, PA1-511A, ThermoFisher). After washing with PBS, sections were incubated for 1 h at room temperature with the following secondary antibodies: goat anti-mouse IgG H&L (Alexa Fluor 488; ab222914) and goat anti-rabbit IgG (H + L) secondary antibody DyLight-488 (#35552, ThermoFisher). Nuclei were stained with DAPI (1/10,000). Fluorescent images were captured using an OLYMPUS DX51 microscope (Tokyo, Japan) and analyzed with DP2-BSW software (version 2.2, Tokyo, Japan) and Image Pro Plus (version 6.0, Media Cybernetics, Rockville, MD, USA). Quantification of Gr-1^+^ and CD68^+^ cells was performed in a blinded manner across five high-power fields per slide for each of the eight animals per group.

### 2.8. Immunohistochemical Staining

Paraffin-embedded liver sections were deparaffinized, rehydrated through a graded ethanol series and distilled water, and treated with 3% H_2_O_2_ in methanol for 30 min to block endogenous peroxidase activity. After two washes in PBS for 5 min each, sections were incubated with 5% goat serum for 30 min to prevent nonspecific antibody binding. After washing, sections were incubated overnight at 4°C with primary antibodies c-CASPASE-3 (1:200, #9661, CST) and Esr1 (1:250, MA1-27107, Invitrogen). After additional PBS washes, sections were incubated with appropriate secondary antibodies. DAB chromogen was applied as per the manufacturer's instructions and images were captured using a digital microscope camera (Nikon). Expression levels were evaluated based on the proportion of positively stained cells (0% = 0; 1%–25% = 1; 26%–50% = 2; 51%–75% = 3; 76%–100% = 4) multiplied by the intensity of staining (0 = negative; 1 = weak; 2 = moderate; 3 = strong). Final scores were determined independently by two pathologists in a blinded manner.

### 2.9. RT-qPCR Analysis

Total RNA was extracted using TRIZOL (Invitrogen, USA) and reverse-transcribed into cDNA using the High-Capacity cDNA Reverse Transcription Kit (Applied Biosystems: 4368813). The reverse transcription conditions were set at 37°C for 15 min, 85°C for 5 s, followed by a hold at 4°C. RT-qPCR was performed using the SYBR Premix Ex Taq (Tli RNaseH Plus) kit (RR820A, TaKaRa, Japan) on an ABI 7500 Real-Time PCR System (Thermo Fisher Scientific, USA). The 25 μL PCR reaction mixture was subjected to the following thermal cycling conditions: initial denaturation at 95°C for 5 min, followed by 40 cycles of 95°C for 10 s, 60°C for 20 s, 72°C for 20 s, and 78°C for 20 s. Data were analyzed using the 2^−ΔΔCt^ method. Glyceraldehyde-3-phosphate dehydrogenase (GAPDH) was used as an internal control. Primer sequences were designed and synthesized by Invitrogen and are listed in Supporting Information 1: Table S3.

### 2.10. Western Blot

Total proteins were extracted using a RIPA lysis buffer (R0010, Solarbio Science & Technology Co., Ltd., Beijing, China) containing 1% protease inhibitor and 1% phosphatase inhibitor (Beyotime, Shanghai, China). Protein concentrations were determined using a BCA protein assay kit (Jet Bio-Filtration Co., Ltd., Guangzhou, China). Equal amounts of protein (40 μg per sample) were separated by 10% SDS-PAGE and transferred to PVDF membranes (Millipore, USA). Membranes were blocked with 5% bovine serum albumin (BSA) in TBST at room temperature, followed by overnight incubation at 4°C with diluted primary antibodies against Bcl2 (1:1000, ab196495, Abcam), Bax (1:2000, ab32503, Abcam), c-CASPASE-3 (1:1000, #9661, CST), CASPASE-3 (1:1000, ab184787, Abcam), Esr1 (1:1000, MA1-27107, Invitrogen), p-TAK1 (1:2000, ab109404, Abcam), TAK1 (1:2000, ab109526, Abcam), p-p38 (1:2000, ab170099, Abcam), p38 (1:2000, ab32142, Abcam), p-JNK (1:2000, ab124956, Abcam), JNK (1:1000, ab179461, Abcam), Nrf2 (1:2000, ab62352, Abcam), HO-1 (1:2000, ab305290, Abcam), NQO1 (1:2000, ab80588, Abcam), and GAPDH (1:2500, ab9485, Abcam). After washing, membranes were incubated at room temperature with HRP-conjugated goat anti-rabbit IgG secondary antibody (1:2000; ab97051, Abcam) on a shaker. Protein bands were visualized using enhanced chemiluminescence (ECL) and imaged with an Image Quant LAS 4000C gel imaging system (GE Healthcare, USA).

### 2.11. Co-Immunoprecipitation (Co-IP)

For the immunoprecipitation assay, proteins were incubated with an anti-ESR1 antibody (1:1000, MA1-27107, Invitrogen) or isotype control IgG (1:2000, ab97051, Abcam). Immunocomplexes were collected and subsequently analyzed by Western blot using a primary antibody against TAK1 (1:2000, ab109526, Abcam). Band detection and imaging were performed using the ImageQuant LAS 4000C imaging system (GE, USA).

### 2.12. Statistical Analysis

All data were processed using SPSS software version 21.0 (SPSS Inc., Chicago, IL, USA). Quantitative data were expressed as mean ± standard deviation. Comparisons between two groups were performed using independent sample *t*-tests, while comparisons among multiple groups were conducted using one-way analysis of variance (ANOVA), followed by Tukey's post hoc test. A *p*-value of less than 0.05 was considered statistically significant.

## 3. Results

### 3.1. EA Decreases Liver Injury in Rats With HIRI

**Figure 1:**
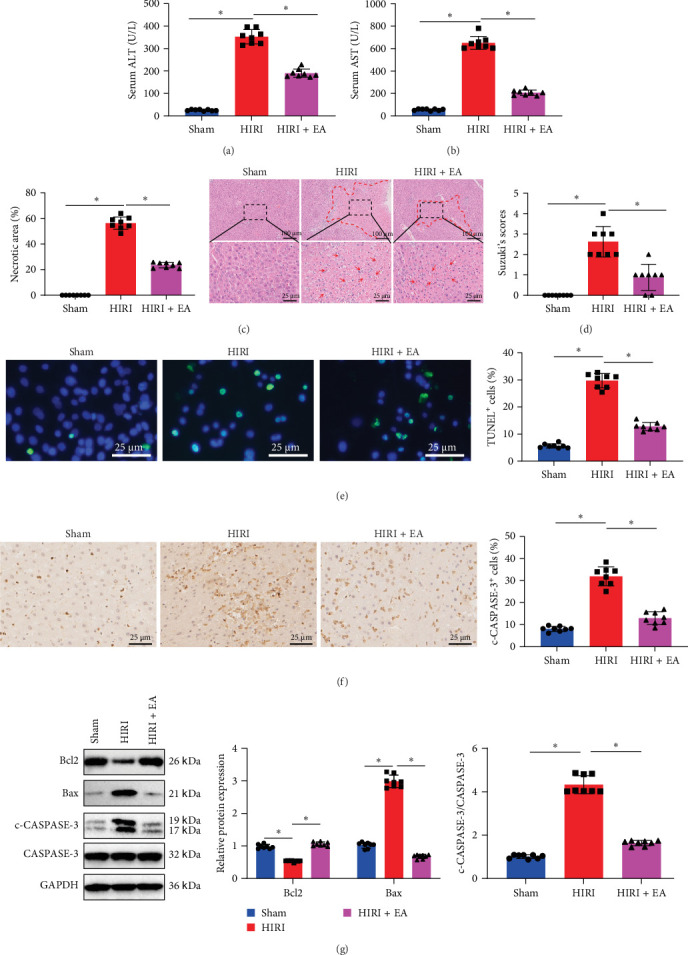
Impact of EA on hepatocyte apoptosis and liver injury in HIRI rats. (A, B) Serum ALT and AST activities were measured in each group. (C) H&E staining revealed necrotic areas in liver tissues (scale bars = 100 and 25 µm), with red arrows indicating necrotic regions; corresponding bar graphs show quantification of necrotic area in each group. (D) Liver injury severity was assessed using Suzuki's scoring system. (E) TUNEL staining identified apoptotic cells in the liver tissues (green: TUNEL-positive; blue: DAPI-stained nuclei; scale bar = 25 µm). (F) Immunohistochemistry for c-CASPASE-3 expression in liver tissues (scale bar = 25 µm). (G) Western blot analysis of Bcl2, Bax, and the ratio of c-CASPASE-3 to CASPASE-3. *⁣*^*∗*^*p*  < 0.05 between groups. One-way ANOVA was used for comparisons among multiple groups, followed by Tukey's post hoc test. Each group included eight rats.

Prior research has indicated that percutaneous EA at specific acupoints can alleviate ischemia-reperfusion injury [20, 28]. To examine the molecular mechanism of EA on the protection of HIRI, a rat model of HIRI was established. The HIRI group exhibited notably elevated serum transaminase ALT and AST levels (Figure 1A,B) and worsened liver injury with increased necrotic area and Suzuki score (Figure 1C,D). Additionally, hepatocyte apoptosis, the number of c-CASPASE-3-positive cells, Bax expression, and the c-CASPASE-3/CASPASE-3 ratio were significantly increased, whereas Bcl2 expression was markedly reduced in liver tissues (Figure 1E,G). These findings suggest the successful establishment of the HIRI model compared to the Sham group.

EA was subsequently administered to rats with HIRI to evaluate its protective effects. Serum biochemical analysis revealed that the HIRI + EA group exhibited significantly lower ALT and AST levels compared to the HIRI group, as illustrated in Figure 1A,B. Histological examination using H&E staining showed reduced hepatic damage in the EA-treated group, as evidenced by smaller necrotic areas and lower Suzuki scores (Figure 1C,D). TUNEL staining further indicated a marked reduction in hepatocyte apoptosis following EA treatment (Figure 1E). Immunohistochemical analysis demonstrated a decrease in the number of c-CASPASE-3-positive cells in the HIRI + EA group relative to the HIRI group (Figure 1F). Western blot analysis revealed increased Bcl2 expression and reduced Bax expression and c-CASPASE-3/CASPASE-3 ratio in liver tissues of the EA-treated group (Figure 1G).

### 3.2. EA Diminishes the Inflammatory Reaction in the Liver Tissue of Rats With HIRI

**Figure 2:**
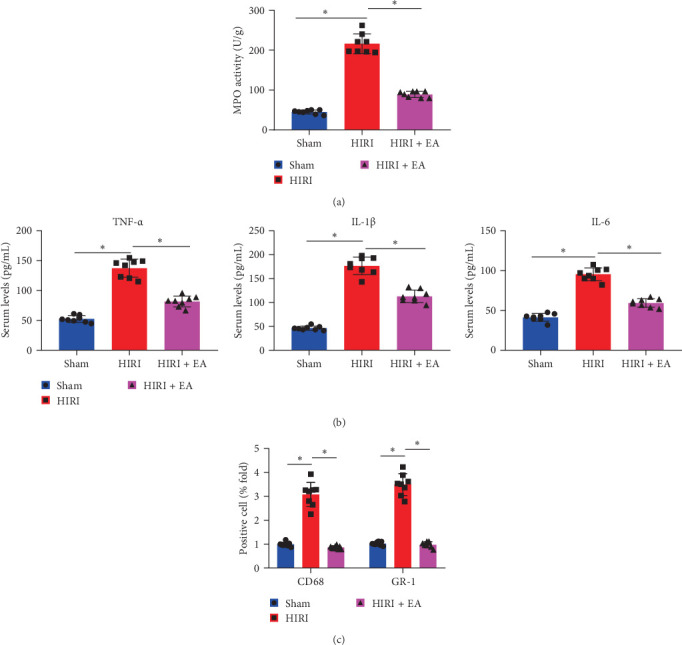
Effects of EA on inflammatory cell infiltration and cytokine release in HIRI rat liver tissues. (A) Myeloperoxidase (MPO) activity in liver tissues of each group. (B) Serum levels of TNF-α, IL-1β, and IL-6 measured by ELISA. (C) Immunofluorescence staining displayed representative images of CD68-positive and GR-1-positive cells in the liver tissues. *⁣*^*∗*^*p*  < 0.05 between groups, analyzed by one-way ANOVA followed by Tukey's post hoc test for multiple groups. Each group consisted of eight rats.

Previous studies have shown that polymorphonuclear cells (PMNs) and other immune cells rapidly accumulate in the liver during the early phase of reperfusion [29]. According to Figure 2A, the liver MPO activity was significantly higher in the HIRI group compared to the Sham group. However, the HIRI + EA group exhibited a significant decrease in liver MPO activity compared to the HIRI group.

ELISA findings indicated that in comparison to the Sham group, the HIRI group exhibited notably elevated TNF-α, IL-1β, and IL-6 levels in the serum of rats. Conversely, the HIRI + EA group displayed significantly decreased TNF-α, IL-1β, and IL-6 serum levels when compared to the HIRI group (Figure 2B). To assess the infiltration of inflammatory cells in the liver tissue of HIRI rats, immunofluorescence staining was performed using GR-1 as a marker for neutrophils and CD68 for macrophages. Compared to the Sham group, the HIRI group exhibited a notable rise in neutrophil and inflammatory macrophage cells, whereas the HIRI + EA group demonstrated a significant decrease in both cell populations relative to the HIRI group (Figure 2C). The results suggest that EA can relieve liver tissue inflammation in rats by decreasing the quantity of inflammatory cells and the secretion of inflammatory substances.

### 3.3. EA Markedly Decreases the Expression of Esr1 in the Liver Tissue of Rats With HIRI

**Figure 3:**
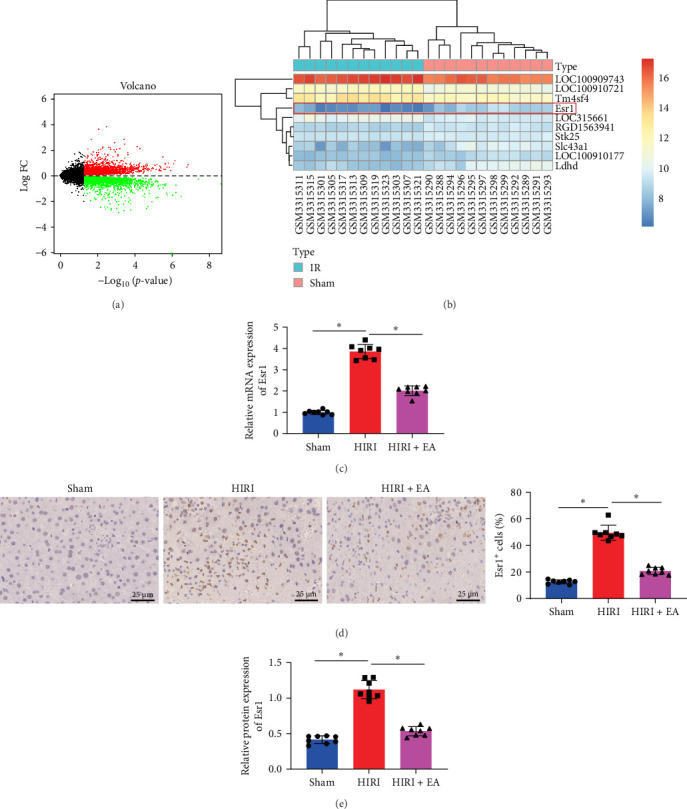
Influence of EA on Esr1 expression in HIRI rat liver tissues. (A) Volcano plot of significantly differentially expressed mRNAs from the GSE117915 dataset. (B) Heatmap of the top 10 differentially expressed genes with the smallest *p*-values. (C) RT-qPCR analysis of Esr1 mRNA expression in each group. (D) Immunohistochemical staining was employed to assess Esr1 levels in the liver tissues (scale bar = 25 µm). (E) Western blot analysis of Esr1 protein levels. All experiments were conducted in triplicate. *⁣*^*∗*^*p*  < 0.05 between groups. Statistical comparisons were made using one-way ANOVA with Tukey's post hoc test. Each group included eight rats.

Differential expression analysis of the HIRI-related microarray dataset GSE117915 identified 5093 DEGs (Figure 3A). The top 10 genes with the most significant *p*-values were LOC100909743, LOC100910721, Tm4sf4, Esr1, LOC315661, RGD1563941, Stk25, Slc43a1, LOC100910177, and Ldhd (Figure 3B).

RT-qPCR was performed to validate the expression levels of the top 10 candidate genes in liver tissues from Sham and HIRI rats. Esr1 expression was significantly elevated in liver tissue from HIRI rats compared to the Sham group, whereas ET-1 levels were markedly reduced in the HIRI + EA group relative to the HIRI group (Figure 3C). In addition, RT-qPCR analysis showed that the remaining nine genes exhibited a notable rise in the mRNA levels in the liver tissue of HIRI rats compared to the Sham group. However, no noticeable distinction was observed between the HIRI and HIRI + EA groups (Supporting Information 2: Figure S1A–I).

Moreover, the Esr1 protein level was evaluated through immunohistochemical staining and western blot analysis. In the liver tissue of HIRI rats, the expression of Esr1 was significantly increased compared to the Sham group. In contrast, Esr1 expression was markedly reduced in the HIRI + EA group relative to the HIRI group, as shown in Figure 3D,E. These results suggest that EA could decrease the expression of Esr1 in the liver tissue of rats with HIRI.

### 3.4. Silencing Esr1 Alleviates Liver Injury in HIRI Rats

Previous studies have discovered that the activation of Esr1 can influence cell apoptosis [30, 31]. Based on these findings, we hypothesize that EA may alleviate HIRI by downregulating the expression of Esr1.

To further examine the influence of Esr1 on HIRI in rat livers, primary hepatocytes were isolated from HIRI rats and examined for morphological characteristics under a light microscope (Supporting Information 3: Figure S2). Adenoviral vectors were employed to introduce sh-Esr1, and the expression of Esr1 was assessed using RT-PCR and western blotting. The findings indicated that sh-Esr1-1 exhibited highest knockdown efficiency and was selected for subsequent in vivo experiments (Supporting Information 4: Figure S3A,B).

**Figure 4:**
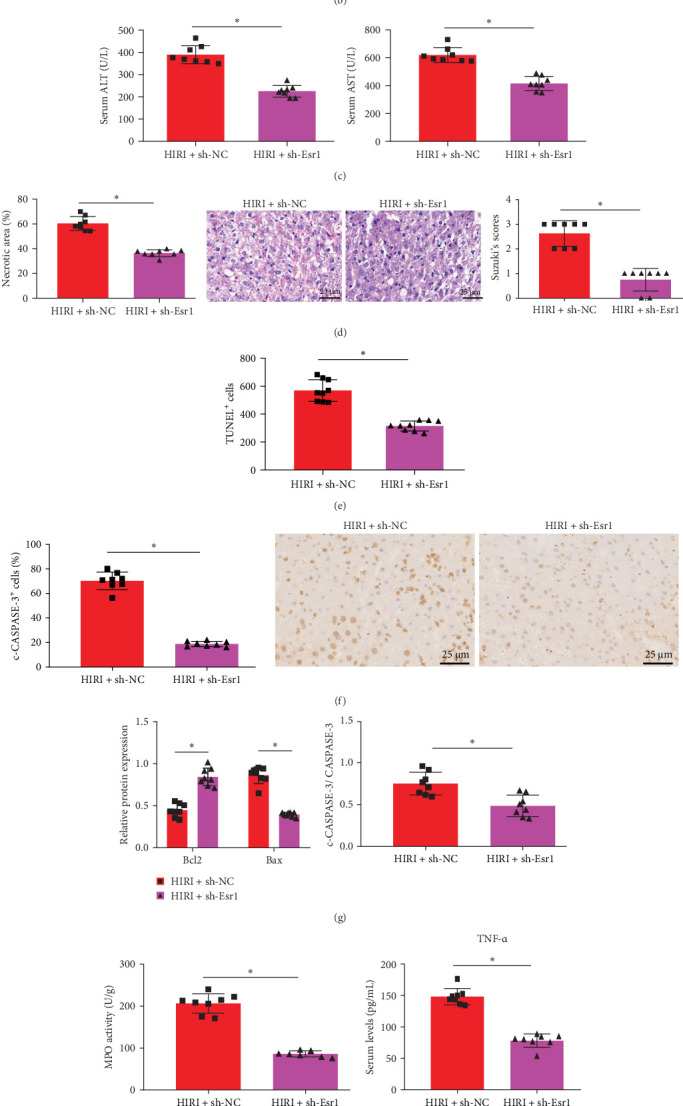
Effects of Esr1 silencing on liver injury in HIRI rats. (A) Immunohistochemical staining of Esr1 in liver tissues (scale bar = 25 μm). (B) Western blot analysis of Esr1 protein expression in liver tissues. (C) Serum ALT and AST levels in each group. (D) H&E staining of liver sections and quantification of necrotic areas; liver injury severity assessed by Suzuki score (scale bar = 25 µm). (E) TUNEL staining to detect apoptotic hepatocytes. (F) Immunohistochemical staining of c-CASPASE-3 in liver tissues (scale bar = 25 μm). (G) Western blot analysis of Bcl2, Bax, and c-CASPASE-3/CASPASE-3 ratio. (H) MPO activity in the liver tissues of each group of rats. (I–K) Serum levels of TNF-α, IL-1β, and IL-6 measured by ELISA. (L) Immunofluorescence staining of CD68^+^ macrophages and GR-1^+^ neutrophils. *⁣*^*∗*^*p*  < 0.05 among groups, analyzed by independent sample *t*-test (two-group comparison). Each group included eight rats.

Esr1 was silenced in vivo and its expression in liver tissue was evaluated by immunohistochemical staining and western blotting. As shown in Figure 4A,B, Esr1 expression in liver tissue was markedly reduced in the HIRI + sh-Esr1 group compared to the HIRI + sh-NC group. The extent of liver injury was assessed by measuring the levels of serum ALT and AST. The results indicated a notable decrease in the HIRI + sh-Esr1 rat group relative to the HIRI + sh-NC group (Figure 4C).

H&E staining demonstrated that the HIRI + sh-Esr1 group had a significant reduction in liver damage compared to the HIRI + sh-NC group, as evidenced by decreased necrotic area and lower Suzuki scores (Figure 4D). As illustrated in Figure 4E, TUNEL staining revealed a notable decrease in hepatocyte apoptosis in the HIRI + sh-Esr1 group compared to the HIRI + sh-NC group, indicating enhanced cell survival following Esr1 silencing. In rat liver tissue, the immunohistochemical staining of c-CASPASE-3 showed a significant decrease in the number of positive cells in the HIRI + sh-Esr1 group compared to the HIRI + sh-NC group (Figure 4F).

In addition, the western blot analysis revealed that the HIRI + sh-Esr1 group exhibited a notable increase in the expression of Bcl2, whereas the expression of Bax and the ratio of c-CASPASE-3/CASPASE-3 were significantly reduced in the liver tissue compared to the HIRI + sh-NC group (Figure 4G). Furthermore, as depicted in Figure 4H, MPO activity was markedly reduced in the HIRI + sh-Esr1 group. ELISA results further demonstrated significant reductions in serum levels of TNF-α, IL-1β, and IL-6 in the HIRI + sh-Esr1 group relative to the HIRI + sh-NC group (Figure 4). To assess the infiltration of inflammatory cells, immunofluorescence was conducted on rat liver tissue using GR-1 and CD68 as markers for neutrophils and macrophages, respectively. A significant decrease in the infiltration of both cell types was observed in the liver tissue of the HIRI + sh-Esr1 group compared to the HIRI + sh-NC group (Figure 4L). These findings suggest that Esr1 inhibition attenuates hepatocyte apoptosis and hepatic inflammation by reducing neutrophil and macrophage infiltration, thereby alleviating the progression of HIRI in rats.

### 3.5. Protective Effect of EA With Electrical Stimulation on Liver Damage in HIRI Rats is Reversed by the Overexpression of Esr1

**Figure 5:**
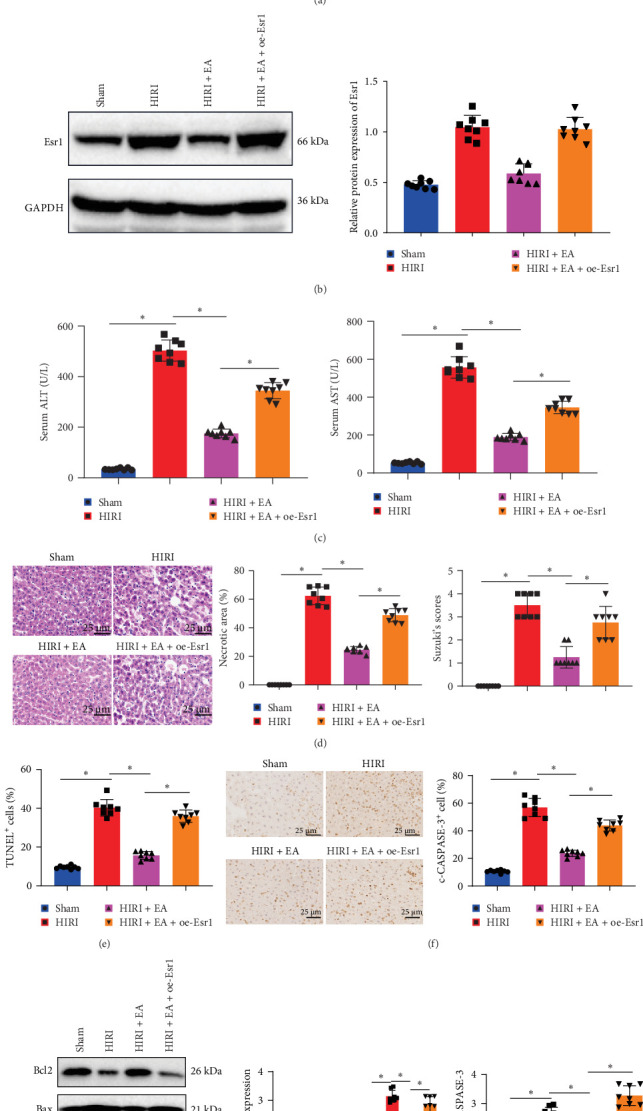
Effects of EA and Esr1 overexpression on HIRI in rats. (A) Immunohistochemical staining of Esr1 in liver tissues (scale bar = 25 μm). (B) Western blot analysis of Esr1 protein levels. (C) Serum ALT and AST activities in each group. (D) H&E staining and Suzuki score evaluation of liver injury severity (scale bar = 25 μm). (E) TUNEL staining to detect apoptotic hepatocytes. (F) Immunohistochemical staining of c-CASPASE-3 ratio (scale bar = 25 μm). (G) Western blot analysis of Bcl2, Bax, and c-CASPASE-3/CASPASE-3 expression. *⁣*^*∗*^*p*  < 0.05 among groups, determined by one-way ANOVA followed by Tukey's post hoc test. Each group consisted of eight rats.

To investigate the protective effects and mechanisms of Esr1 overexpression in the context of EA treatment for HIRI, EA and Esr1 overexpression were simultaneously administered to HIRI rats. The presence of Esr1 in the liver tissues of rats was identified through immunohistochemical staining and western blot analysis. The findings indicated that the level of Esr1 expression was considerably higher in the HIRI + oe-NC group compared to the Sham group. Moreover, the HIRI + EA + oe-NC group exhibited a notably reduced level of Esr1 expression compared to the HIRI + oe-NC group. In contrast, Esr1 levels were significantly elevated in the HIRI + EA + oe-Esr1 group compared to the HIRI + EA + oe-NC group (Figure 5A,B).

To assess liver injury, serum levels of ALT and AST were measured. The HIRI + oe-NC group exhibited markedly elevated levels of AST and ALT compared to the Sham group. Furthermore, the HIRI + EA + oe-NC group exhibited notably reduced levels of AST and ALT compared to the HIRI + oe-NC group. In contrast, the HIRI + EA + oe-Esr1 group exhibited notably elevated levels of AST and ALT compared to the HIRI + EA + oe-NC group (Figure 5C).

We also assessed the extent of liver damage in rats using H&E and TUNEL staining. Compared to the Sham group, the HIRI + oe-NC group exhibited markedly aggravated liver damage, as indicated by increased necrotic areas and elevated Suzuki scores. In contrast, the HIRI + EA + oe-NC group showed a notable reduction in liver damage, with reduced necrotic regions and lower Suzuki scores. However, liver injury was notably worsened in the HIRI + EA + oe-Esr1 group compared to the HIRI + EA + oe-NC group (Figure 5D,E).

Immunohistochemical staining of c-CASPASE-3 in rat liver tissues revealed a notable rise in the distribution of c-CASPASE-3 expression in the HIRI + oeNC group compared to the Sham group. Conversely, the HIRI + EA + oeNC group exhibited a significant decrease in the distribution of c-CASPASE-3 expression compared to the HIRI + oeNC group. Additionally, the HIRI + EA + oeEsr1 group displayed a significant increase in the distribution of c-CASPASE-3 expression in comparison to the HIRI + EA + eNC group (Figure 5F). Western blot analysis revealed a significant decrease in Bcl2 expression in the HIRI + EA + oeNC group compared to the Sham group. Additionally, the HIRI + EA + oe-Esr1 group exhibited a significant increase in Bax expression and c-CASPASE-3/CASPASE-3 values compared to the HIRI + EA + oe-NC group. Additionally, Bcl2 expression in the HIRI + oe-NC group was significantly higher than in the HIRI group, while Bax expression and the c-CASPASE-3/CASPASE-3 ratio were significantly lower Conversely, the Bcl2 expression in the HIRI + EA + oe-Esr1 group exhibited a significant decrease compared to the HIRI + oe-NC group, along with a significant decrease in the Bax expression and c-CASPASE-3/CASPASE-3 values. The levels of CASPASE-3/CASPASE-3 were notably higher in the HIRI + EA/Esr1 group when compared to the HIRI + EA/Esr1 group, as shown in Figure 5G. The findings indicate that Esr1 overexpression counteracts the protective effects of EA on liver damage in rats with HIRI.

### 3.6. EA Mitigates Liver Damage in Rats With HIRI by Suppressing the Expression of Esr1 and Hindering the Activation of the TAK1–JNK/p38 Pathway

**Figure 6:**
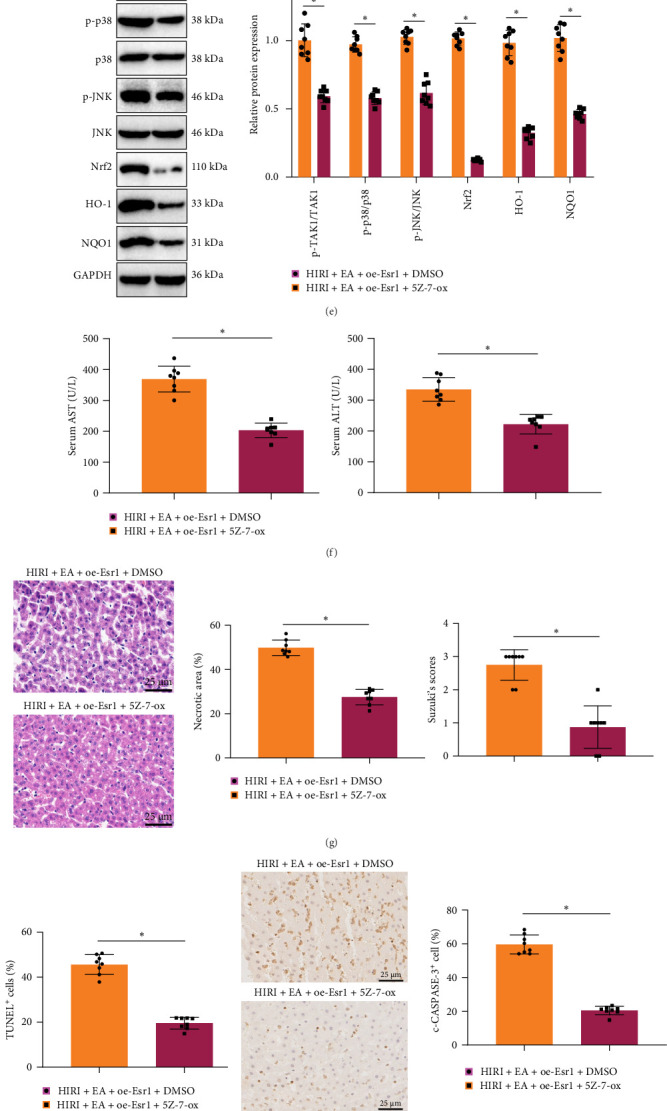
EA modulates HIRI via the Esr1/TAK1-JNK/p38 axis. (A) MPO activity in liver tissues. (B) Serum levels of TNF-α, IL-1β, and IL-6 measured by ELISA. (C) Immunofluorescence staining of CD68^+^ macrophages and GR-1^+^ neutrophils. (D) Western blot analysis of of p-TAK1, p-JNK, p-p38, Nrf2, HO-1, and NQO1 in liver tissues from Sham, HIRI + oe-NC, HIRI + EA + oe-NC, and HIRI + EA + oe-Esr1 groups. (E) Western blot analysis of TAK1–JNK/p38 signaling pathway-related proteins and Nrf2, HO-1, and NQO1 levels in HIRI + EA + oe-Esr1 rats treated with DMSO or TAK1 inhibitor 5 Z-7-ox. (F) Serum ALT and AST levels in each group. (G) H&E staining was used to detect the necrotic areas of the liver and Suzuki's score was used to evaluate liver injury severity in each group (scale bar = 25 μm). (H) TUNEL staining was used to detect cell apoptosis in liver tissues of rats in each group. (I) Immunohistochemical staining for c-CASPASE-3 expression in liver tissues (scale bar = 25 μm). (J) Western blot analysis of Bcl2, Bax, and the c-CASPASE-3/CASPASE-3 ratio. (K) MPO activity measured in liver tissues. (L) LISA assessment of serum TNF-α, IL-1β, and IL-6 levels. (M) Immunofluorescence staining of CD68^+^ macrophages and GR-1^+^ neutrophils in liver tissues. (N) Co-immunoprecipitation (CO-IP) analysis to detect the interaction between ESR1 and TAK1. *⁣*^*∗*^*p*  < 0.05 among groups, analyzed by one-way ANOVA followed by Tukey's post hoc test. Each group comprised eight rats.

To elucidate the molecular mechanism by which EA exerts anti-inflammatory effects in HIRI, additional experiments were conducted under conditions of Esr1 overexpression. As shown in Figure 6A, the liver MPO activity of rats in the HIRI + oe-NC group exhibited a significant increase when compared to the Sham group. The liver MPO activity of rats in the HIRI + EA + oe-NC group showed a significant decrease in comparison to the HIRI + oe-NC group. However, Esr1 overexpression reversed this effect, resulting in a marked increase in MPO activity in the HIRI + EA + oe-Esr1 group. Consistent with these findings, ELISA analysis revealed that serum levels of TNF-α, IL-1β, and IL-6 in the HIRI + oe-NC group exhibited a significant rise when compared to the Sham group. The serum concentrations of TNF-α, IL-1β, and IL-6 in rats were significantly reduced in the HIRI + EA + oe-NC group compared to the HIRI + oe-NC group. In contrast, these cytokine levels were significantly elevated in the HIRI + EA + oe-Esr1 group relative to the HIRI + EA + oe-NC group (Figure 6B). Immunofluorescence analysis demonstrated a significant increase in the number of inflammatory cells, including neutrophils and macrophages, in the HIRI + oe-NC group compared to the Sham group. The HIRI + EA + oe-NC group exhibited a significant reduction in the infiltration of inflammatory cells, including neutrophils and macrophages, compared to the HIRI + oe-NC group. In contrast, the HIRI + EA + oe-Esr1 group exhibited a notable rise in neutrophil and macrophage infiltration relative to the HIRI + EA + oe-NC group (Figure 6C).

The TAK1–JNK/p38 signaling pathway plays a critical role in hepatocellular injury, and inhibition of TAK1 has been shown to alleviate HIRI by inhibiting the subsequent JNK/p38 signaling pathway [32]. To determine whether Esr1 regulates HIRI through this pathway, Western blot analysis was performed. Compared with the Sham group, the expression levels of p-TAK1, p-JNK, and p-p38 were significantly increased in the liver tissues of rats in the HIRI + oe-NC group. In contrast, their expression levels were markedly reduced in the HIRI + EA + oe-NC group compared to the HIRI + oe-NC group. Furthermore, compared to the HIRI + EA + oe-NC group, the HIRI + EA + oe-Esr1 group exhibited elevated expression of p-TAK1, p-JNK, and p-p38. In addition, western blot analysis revealed that EA markedly upregulated the expression of hepatoprotective proteins (Nrf2, HO-1, and NQO1), suggesting that EA may also exert protective effects through activation of additional cytoprotective pathways (Figure 6D). The findings suggest that Esr1 triggered the TAK1–JNK/p38 signaling pathway in the liver tissue of rats with HIRI.

To further investigate whether EA confers hepatoprotection in HIRI rats through modulation of the Esr1/TAK1–JNK/p38 axis, we coadministered the TAK1 inhibitor 5Z-7-ox during EA treatment and Esr1 overexpression. Western blot results showed that compared with the HIRI + EA + oe-Esr1+DMSO group, the HIRI + EA + oe-Esr1 + 5Z-7-ox group exhibited markedly reduced expression of p-TAK1, p-JNK, and p-p38, as well as decreased expression of Nrf2, HO-1, and NQO1 proteins (Figure 6E). Furthermore, serum biochemical analysis revealed that AST and ALT activities were significantly decreased in the HIRI + EA + oe-Esr1 + 5Z-7-ox group compared to the HIRI + EA + oe-Esr1+DMSO group (Figure 6F).

H&E staining revealed a significant reduction in liver damage in the HIRI + EA + oe-Esr1 + 5Z-7-ox group compared to the HIRI + EA + oe-Esr1 + DMSO group, as evidenced by decreased necrotic areas and lower Suzuki scores (Figure 6G).TUNEL staining further demonstrated that hepatocyte apoptosis was markedly reduced in the HIRI + EA + oe-Esr1 + 5Z-7-ox group (Figure 6H).

Immunohistochemical staining of c-CASPASE-3 showed a notable reduction in the expression and distribution of c-CASPASE-3 in the HIRI + EA + oe-Esr1 + 5Z-7-ox group when compared to the HIRI + EA + oe-Esr1+DMSO group (Figure 6I). As shown in Figure 6J, the western blot analysis results indicated that the Bcl2 expression in the liver tissue of rats in the HIRI + EA + oe-Esr1 + 5Z-7-ox group showed a significant increase compared to the HIRI + EA + oe-Esr1+DMSO group. Additionally, the expression of Bax and the c-CASPASE-3/CASPASE-3 value exhibited a significant decrease.

As illustrated in Figure 6K, MPO activity was significantly decreased in the HIRI + EA + oe-Esr1 + 5Z-7-ox group compared to the HIRI + EA + oe-Esr1+DMSO group. ELISA results indicated that the levels of TNF-α, IL-1β, and IL-6 were significantly reduced in the HIRI + EA + oe-Esr1 + 5Z-7-ox group compared to the HIRI + EA + oe-Esr1+DMSO group (Figure 6L). Immunofluorescence analysis revealed a significant decrease in the number of inflammatory cells, including neutrophils and macrophages, in the HIRI + EA + oe-Esr1 + 5Z-7-ox group relative to the HIRI + EA + oe-Esr1+DMSO group (Figure 6M). To determine whether ESR1 directly interacts with TAK1, Co-IP was performed. The results confirmed a physical interaction between ESR1 and TAK1 in liver tissue (Figure 6N).

These findings indicate that EA at the Esr1-reversal acupoint provides protection against HIRI by suppressing the Esr1 expression and subsequently inhibiting the activation of the TAK1–JNK/p38 pathway.

## 4. Discussion

The present study demonstrates that EA at specific acupoints can mitigate hepatocyte apoptosis and inflammation in rats with HIRI, thus, alleviating liver damage. These findings suggest the potential of EA as a therapeutic approach for liver-related diseases. Similar effects have been reported in models of hepatic fibrosis, where EA at the Hegu (LI4) and Taichong (LR3) acupoints improved liver function and reduced liver damage in rats with hepatic fibrosis [33, 34]. Additionally, studies have shown that stimulation of the Zusanli (ST36) and Neiguan (PC6) acupoints can enhance liver function and reduce inflammation in rats with nonalcoholic fatty liver disease [35]. Although these studies primarily address liver fibrosis and target different acupoints, the present research provides new evidence supporting EA as a specific therapeutic strategy for HIRI, thereby contributing to the development of targeted interventions for acute liver injury.

Furthermore, our experimental results indicate that EA can inhibit the Esr1/TAK1–JNK/p38 signaling pathway, thereby reducing hepatocyte apoptosis and inflammatory responses and ultimately mitigating liver damage in HIRI rats. These findings suggest that EA may serve as an effective treatment for HIRI in rats. In addition to liver-related conditions, previous studies have highlighted the therapeutic potential of EA in neurological and cardiovascular diseases [36]. This study is among the first to confirm the efficacy of EA in HIRI and to elucidate its underlying molecular mechanisms [37]. Therefore, this research provides strong evidence for the therapeutic applications of EA and offers important guidance for the development of novel treatment strategies for HIRI.

Further results suggest that EA can downregulate Esr1 expression, thereby inhibiting the activation of the TAK1–JNK/p38 pathway and playing a significant role in mitigating liver damage. This finding indicates that EA at specific acupoints can modulate intracellular signaling pathways to exert therapeutic effects. Previous studies have also reported the regulatory influence of EA on the TAK1–JNK/p38 pathway [38] and its impact on autophagy [39]. In contrast to previous studies, the present research specifically focuses on liver injury and its underlying mechanisms, while further elucidating the regulatory effects of EA on intracellular signaling pathways, providing a potential theoretical basis for EA treatment of liver diseases.

In addition to its activation in rats, p38 MAPK is also a critical component in HIRI in mice. Mouse models have shown that p38 aggravates hepatocellular injury by enhancing inflammatory cytokine production, apoptosis, and oxidative stress [40, 41]. However, in rats, p38 may exhibit context-dependent or dual roles, potentially contributing to stress adaptation or protection during specific phases of reperfusion. These species-specific differences may arise from divergent immune cell responses, hepatic microcirculation, and signaling kinetics. Thus, although EA inhibits p38 activation to protect the liver in rats, the broader roles of p38 across models suggest it may not universally exert deleterious effects and must be interpreted with caution.

Moreover, mice are more frequently employed in HIRI research due to their genetic manipulability and well-established knockout systems. However, rats offer greater anatomical fidelity to human liver physiology, particularly in terms of vascular architecture and hemodynamic properties. Rats also provide better model stability for surgical procedures, allowing for more reliable evaluation of therapeutic interventions [42–44]. The use of rat models, as in this study, therefore remains highly appropriate for evaluating translational interventions such as EA.

Importantly, the MAPK pathway, including JNK/p38, often functions in tandem with NF-κB signaling during IRI. TAK1, as an upstream kinase, can simultaneously activate both axes, promoting cytokine transcription and neutrophil infiltration [42, 43]. Although this study did not directly evaluate NF-κB activation, the observed decrease in inflammatory cytokines following EA suggests a potential inhibitory effect on NF-κB as well. Future studies should examine p65 nuclear translocation and IκBα degradation to confirm the extent of NF-κB involvement and its interaction with the TAK1–JNK/p38 pathway in this model.

**Figure 7:**
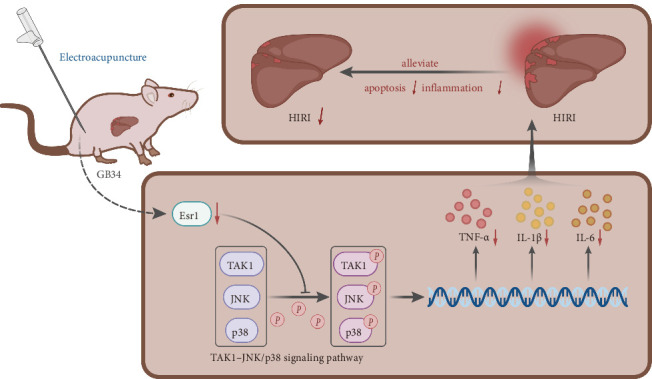
Schematic diagram of the molecular mechanism underlying alleviatory effects of EA on HIRI through the Esr1/TAK1–JNK/p38 axis.

In summary, the findings suggest that EA attenuates hepatocyte apoptosis, suppresses inflammatory cytokine release, and reduces inflammatory cell infiltration in liver tissue, thus, alleviating rat HIRI (Figure 7). The clinical value of this study lies in revealing the protective effects and molecular mechanisms of EA against HIRI, particularly through its regulation of Esr1 and the TAK1–JNK/p38 signaling pathway. Additionally, the potential convergence of TAK1-mediated MAPK and NF-κB signaling in hepatic inflammation provides a promising target for future multipathway therapeutic strategies. These insights offer new directions and strategies for treating HIRI and provide a more scientific and feasible basis for EA's clinical application.

Despite the findings of this study demonstrating that EA alleviates HIRI in rats by downregulating Esr1 expression and inhibiting activation of the TAK1–JNK/p38 signaling pathway, several limitations remain. First, the precise molecular mechanism by which EA regulates Esr1 has not been fully elucidated. Previous studies have shown that ESR1 regulates various downstream pathways, including the ERK1/2 pathway, which exerts protective effects by reducing apoptosis, promoting hepatocyte regeneration, and attenuating inflammation [45–47]. These findings are consistent with our observation that EA-mediated downregulation of Esr1 results in reduced hepatocyte apoptosis and inflammatory response. Therefore, ERK1/2 signaling may also contribute to the protective effects of EA in HIRI, although this hypothesis has not yet been directly investigated and will be a focus of future studies. Second, TAK1 is a central hub in multiple signaling cascades beyond JNK/p38. EA-induced Esr1 suppression may influence other TAK1-dependent pathways, potentially resulting in diverse phenotypic outcomes. Further studies are required to delineate the involvement of alternative downstream signaling pathways in mediating the effects of EA. In addition, this study was conducted exclusively in a rat model of HIRI. Given that Kupffer cells are the primary macrophage population in the liver and play key roles in immune regulation, future studies focusing on Kupffer cells may provide more representative and translationally relevant insights [13, 21]. Compared with rats, mice exhibit lower ischemic tolerance, allowing for more prominent pathological observations. Moreover, generating Esr1 gene mutants in mice could facilitate more detailed mechanistic studies of ESR1 in the context of HIRI [48]. Finally, although the 70% HIRI rat model is a widely accepted experimental tool in liver transplantation research, it does not fully replicate the clinical scenario and has inherent limitations. While this model reproduces key pathological processes observed in clinical liver transplantation, such as ATP depletion and mitochondrial dysfunction during ischemia, ROS burst and sterile inflammation during reperfusion [49, 50], and elicits pro-inflammatory responses (e.g., TNF-α and IL-6) comparable to DAMP-induced innate immune activation in patients [51], anatomical and physiological differences remain. Notably, rats lack a gallbladder and exhibit distinct bile metabolism compared to humans, potentially underestimating the risk of biliary complications such as bile duct strictures [52]. Moreover, the complexity of human immune responses involving regulatory T cells, NK cells, and complement components cannot be fully recapitulated in rat models [53]. Therefore, further validation in diverse animal models and clinical settings is required to confirm the long-term efficacy and safety of this approach.

## Data Availability

The datasets generated and/or analyzed during the current study are available from the corresponding author upon reasonable request.

## Supporting Information

Supporting Information 1 Table S1. Interference sequences. Table S2. Suzuki criteria for grading the degree of liver injury. Table S3. Primer sequences for RT-qPCR. ESR1, estrogen receptor 1; GAPDH, glyceraldehyde-3-phosphate dehydrogenase; RT-qPCR, reverse transcription-quantitative polymerase chain reaction.

Supporting Information 2 Figure S1. RT-qPCR assessment of mRNA levels in rat liver tissues. (A) LOC100909743; (B) LOC100910721; (C) Tm4sf4; (D) LOC315661; (E) RGD1563941; (F) Stk25; (G) Slc43a1; (H) LOC100910177; (I) Ldhd. *⁣*^*∗*^*p* < 0.05 among groups, analyzed by one-way ANOVA followed by Tukey's post hoc test.

Supporting Information 3 Figure S2. Morphological images of primary hepatocytes.

Supporting Information 4 Figure S3. Efficiency test for sh-Esr1 silencing in rat hepatocytes. (A) RT-qPCR analysis of Esr1 mRNA levels following transfection with different sh-Esr1 constructs. (B) Western blot analysis of Esr1 protein expression. *⁣*^*∗*^*p* < 0.05 vs. sh-NC control, analyzed by one-way ANOVA. All experiments were independently repeated three times.
