# Supplementary material for: Electroacupuncture Attenuates Hepatic Ischemia-Reperfusion Injury by Modulating the Esr1/TAK1–JNK/p38 Signaling Pathway in Rats
Source: Mediators Inflamm. 2025 Aug 30;2025:4932970. doi: 10.1155/mi/4932970 (PMC12413952; doi:10.1155/mi/4932970)
Supplement: Supporting Information 1 — Table S1. Interference sequences. Table S2. Suzuki criteria for grading the degree of liver injury. Table S3. Primer sequences for RT-qPCR. ESR1, estrogen receptor 1; GAPDH, glyceraldehyde-3-phosphate dehydrogenase; RT-qPCR, reverse transcription-quantitative polymerase chain reaction. [file 4932970.f1.docx]

**Table S1** Interference sequences

| shRNA | Sequences |
| --- | --- |
| sh-Esr1-1 | CCGCCTTCTACAGGTCTAATT |
| sh-Esr1-2 | CCCATGATCTATTCTGAATAT |
| sh-Esr1-3 | GCCGAAATGAAATGGGTGCTT |
| sh-NC | GCTCAACAGTATGGAGGGAAT |

**Table S2 Suzuki criteria for grading the degree of liver injury**

| Score | Congeston | Vacuole degeneration | Necrosis |
| --- | --- | --- | --- |
| 0 | None | None | None |
| 1 | Slight | Slight | Single cell |
| 2 | Mild | Mild | < 30% |
| 3 | Moderate | Moderate | 31-60% |
| 4 | Severe | Severe | > 60% |

**Table S3** Primer sequences for RT-qPCR

| Genes | Sequences |
| --- | --- |
| ESR1 | Forward: 5’-CACTCGATCATTCGAGCACA-3’ |
|  | Reverse: 5’-TTTGGTGTGAAGGGTCATGG-3’ |
| GAPDH | Forward: 5’-CTAGAGACAGCCGCATCTTC-3’ |
|  | Reverse: 5’-CGTTGATGGCAACAATGTCC-3’ |

Note: ESR1, estrogen receptor 1; GAPDH, glyceraldehyde-3-phosphate dehydrogenase; RT-qPCR, reverse transcription-quantitative polymerase chain reaction.
